# Resolving bundled microtubules using anti-tubulin nanobodies

**DOI:** 10.1038/ncomms8933

**Published:** 2015-08-11

**Authors:** Marina Mikhaylova, Bas M. C. Cloin, Kieran Finan, Robert van den Berg, Jalmar Teeuw, Marta M. Kijanka, Mikolaj Sokolowski, Eugene A. Katrukha, Manuel Maidorn, Felipe Opazo, Sandrine Moutel, Marylin Vantard, Frank Perez, Paul M. P. van Bergen en Henegouwen, Casper C. Hoogenraad, Helge Ewers, Lukas C Kapitein

**Affiliations:** 1 Division of Cell Biology, Department of Biology, Faculty of Science, Utrecht University, Padualaan 8, Utrecht 3584 CH, The Netherlands; 2 RG Neuroplasticity, Leibniz Institute for Neurobiology, Brenneckestr. 6, Magdeburg 39118, Germany; 3 Randall Division of Cell and Molecular Biophysics, King's College London, Guy's Campus, London SE1 1UL, UK; 4 Department of Neuro- and Sensory Physiology, University of Göttingen, Humboldtallee 23, Göttingen 37073, Germany; 5 Institut Curie, Research Center, 26, rue d'Ulm, Paris, F-75248, France; 6 CNRS UMR144, 26, rue d'Ulm, Paris, France; 7 Translational Research Department, Institut Curie, 26, rue d'Ulm, Paris F-75248, France; 8 Inserm, U836, F-38000, Grenoble, France. Univ. Grenoble Alpes, Grenoble Institut des Neurosciences, Grenoble F-38000, France

## Abstract

Microtubules are hollow biopolymers of 25-nm diameter and are key constituents of the cytoskeleton. In neurons, microtubules are organized differently between axons and dendrites, but their precise organization in different compartments is not completely understood. Super-resolution microscopy techniques can detect specific structures at an increased resolution, but the narrow spacing between neuronal microtubules poses challenges because most existing labelling strategies increase the effective microtubule diameter by 20–40 nm and will thereby blend neighbouring microtubules into one structure. Here we develop single-chain antibody fragments (nanobodies) against tubulin to achieve super-resolution imaging of microtubules with a decreased apparent diameter. To test the resolving power of these novel probes, we generate microtubule bundles with a known spacing of 50–70 nm and successfully resolve individual microtubules. Individual bundled microtubules can also be resolved in different mammalian cells, including hippocampal neurons, allowing novel insights into fundamental mechanisms of microtubule organization in cell- and neurobiology.

## Abstract (web summary)

Super-resolution imaging of microtubules requires labels that increase their apparent diameter, making it difficult to resolve individual microtubules within a bundle. Here, the authors develop single-chain antibody fragments against tubulin that enable closely spaced individual microtubules to be distinguished in cells.

## Graphical abstract

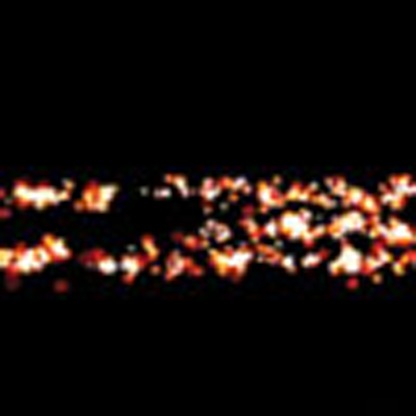

Microtubules are hollow biopolymers of 25-nm diameter and are key constituents of the cellular cytoskeleton, the mechanical framework of dynamic polymers and associated proteins that directs cell shape and facilitates intracellular transport^1^. The exact spatial organization of microtubules and their bundling is of central importance to a number of fundamental cellular processes such as mitosis, cell polarization and the outgrowth of cellular processes, for example, in neurons^1^. Conventional fluorescence microscopy allows selective labelling of microtubule modifications and associated proteins, but cannot resolve individual microtubules within tightly bundled microtubule arrays. Electron microscopy, in contrast, allows resolving individual microtubules, but is very labour intensive, while high-density labelling of specific proteins has remained challenging. Single-molecule localization microscopy (SMLM) provides selectivity at an increased resolution, but the extremely small spacing between neuronal microtubules (20–70 nm)^2^ poses novel challenges, because existing labelling strategies typically increase the apparent microtubule diameter by 20–40 nm and will thereby blend neighbouring microtubules into one structure^3^. It is therefore widely assumed that despite all progress in super-resolution microscopy, electron microscopy is still the only technique that allows insight into complex microtubule structures^4^. Here, we use both computer simulations and experimental approaches to explore how labelling strategy affects SMLM imaging of microtubules. We develop single-chain antibody fragments (nanobodies) against tubulin and achieve super-resolution imaging of microtubules with a decreased apparent diameter, allowing us to optically resolve bundled microtubules.

## Results

### Simulations of microtubules with different labels

**Figure 1:**
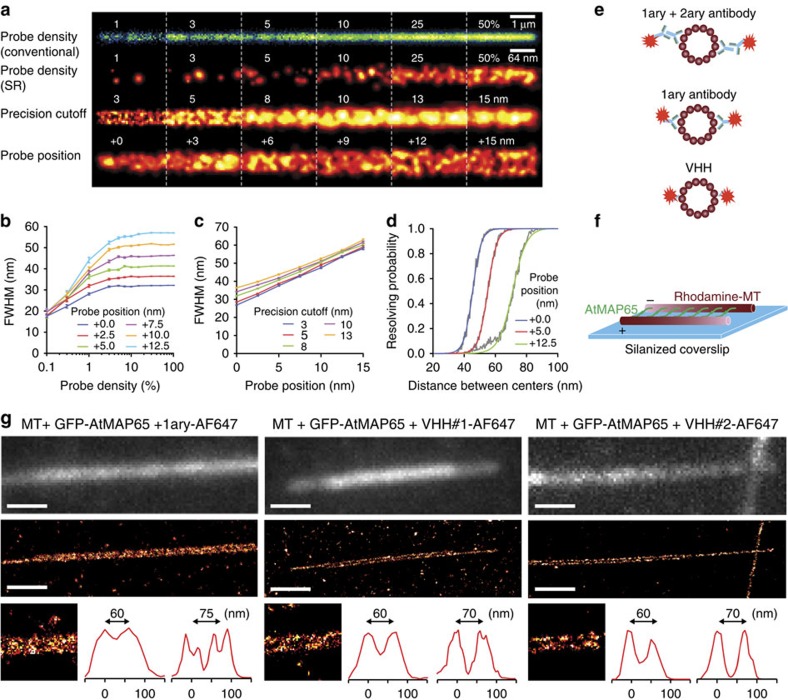
Smaller labels allow resolving bundled microtubules. (**a**) Simulations of conventional (top) and single-molecule localization-based microtubule images for different probe densities, localization precision cutoffs and probe positions (distance between target molecule and fluorophore). Unless specified otherwise, probe position is 2.5 nm and precision cutoff is 8 nm. Probe density is 100% and 50% for the third and fourth row, respectively. A Gaussian localization accuracy distribution with mean±s.d. of 7.5±2.5 nm is used. (**b**) FWHM of Gaussian fits to microtubule cross sections integrated over 512 nm length as a function of probe density and for different probe positions. Error bars represent s.e.m. Each point is the average of 150 FWHMs measured on 512 nm long microtubule (MT; empty stretches along the MT were not included). (**c**) MT FWHM versus probe position for different cutoffs of the localization accuracy distribution. (**d**) Estimation of resolving power for staining of microtubules with probes at increasing distance from the microtubule. Probe density is 7%, localization precision cutoff threshold is 13 nm. Two-hundred and fifty profiles per distance. (**e**) Illustration of the different labelling strategies compared in this study. (**f**) Scheme of the *in vitro* microtubule bundling assay to test the resolving power of different microtubule labelling strategies. Rhodamine-labelled microtubules are assembled into planar bundles with defined spacing formed by the microtubule-bundler GFP–AtMAP65-1. (**g**) Conventional (top) and SMLM (middle and bottom left) images and representative line scans (bottom right) of *in vitro* microtubule bundles stained with a fluorescently labelled primary anti-α-tubulin antibody (1ary-AF647) or two novel tubulin nanobodies (VHH#1 and VHH#2) conjugated to AF647. Scale bar, 1 μm. More examples are provided in Supplementary Fig. 3.

To explore the effect of label size and fluorescent probe positioning on resolving ability, we first performed numerical simulations to examine how labelling density, localization precision and fluorophore positioning affect the apparent microtubule width (determined as the full width at half maximum (FWHM) from Gaussian fits to intensity profiles integrated over 512 nm of microtubule length; Fig. 1a). Using a maximum localization uncertainty of 8 nm, we found that the apparent microtubule width was ∼31 nm for a fluorophore positioned directly at the microtubule surface (probe position of 0 nm, Fig. 1b). Placing the fluorophore further away increased the FWHM by double the displacement, that is, 41 nm for a fluorophore position of 5 nm. A more stringent precision cutoff resulted in decreased FWHM (Fig. 1c) and the FWHM decreased from 63 nm for a probe position of 15 nm and precision cutoff at 13 nm to 27 nm with fluorescent probes directly on the microtubule lattice and a precision cutoff of 3 nm.

To examine how label size affects the probability of resolving closely spaced microtubules, pairs of randomly picked profiles were superimposed with a set distance between the microtubule centres and the resulting profile was analysed. If the lowest intensity between the two microtubule centres was <75% of the intensity of the lowest peak, then the microtubules were considered to be resolved and the resolving probability was calculated as the fraction of resolvable cases out of 250. As expected, decreasing label size results in increasing the resolving probability (Fig. 1d). For example, given a labelling density of 7% and a precision cutoff of 13 nm, the probability of resolving microtubules with centres spaced 55-nm apart increased from 0.03 to 0.49 to 0.97 for probes positioned at 12.5 nm, 5 nm and 0 nm from the microtubule lattice, respectively (data taken from fit).

### Generation and characterization of tubulin nanobodies

Conventional staining strategies often use a combination of primary antibodies binding a specific epitope, followed by a fluorescently tagged secondary antibody that recognizes the primary antibody, resulting in significant displacement of the fluorescent probe from the target (Fig. 1e). Typically, smaller labels have been obtained by directly conjugating a fluorophore to the primary antibody, or by using antibody fragments. Antibody fragments derived from heavy chain only camelid antibodies (nanobodies) are now emerging as promising alternatives, because of their small size (∼15 kDa, ∼4 nm), as well as ease of selection and production. Previous work has demonstrated the usage of nanobodies to create smaller labels for SMLM. Overexpression of GFP–tubulin and subsequent labelling with an anti-GFP nanobody conjugated to a fluorescent dye significantly decreased the effective diameter of individual microtubules^3^. However, this strategy requires overexpression of GFP–tubulin to very high levels, which will perturb cytoskeletal organization and is not possible in many biological systems.

To experimentally assess the effect of label size on resolving power, we created three novel labels for SMLM of endogenous tubulin, complementing the existing strategies using conventional antibodies. First, we developed two different nanobodies against tubulin. One was derived from two rounds of phage display selection using a universal synthetic library of humanized nanobodies (VHH#1) and the other using an MRC7 cell library (VHH#2) (Supplementary Fig. 1a,b; see Methods section for details), similarly selected in two rounds of phage display. Immunoblotting with VHH#1 or VHH#2 on lysates of HEK293 cells overexpressing GFP–α-tubulin or GFP–β-tubulin revealed that both nanobodies react with the endogenous tubulin as well as GFP–β-tubulin (Supplementary Fig. 2a,b). Conjugation of Alexa Fluor 647 (AF647) to the nanobodies did not interfere with their binding properties (Supplementary Fig. 2c; Supplementary Figs 3 and 4). As a second approach, recombinant human-derived single-chain variable fragments (scFvs) directed against α- and β-tubulin were purified and also coupled to AF647 (ref. 5). All bacterially expressed and purified labels were relatively pure and stable over long periods of time (Supplementary Fig. 2a).

### Resolving microtubule bundles *in vitro*

To test the SMLM resolving power of the different microtubule labels, we established an *in vitro* bundling assay using polymerized microtubules in combination with the microtubule bundler AtMAP65-1, which promotes the formation of a planar network of antiparallel microtubules with a single-dimer spacing in between (Fig. 1f)^6^. Silanized coverslips were used to stably attach the microtubule bundles to the coverslip surface to allow for subsequent staining procedures. As a control, we performed SMLM on non-stained samples to which fluorescently tagged tubulin (conjugated to HiLyte Fluor 647) was added in to the polymerization mix. In this condition, most bundles could be clearly resolved with an average spacing of 65±2 nm (s.e.m., *n*=56, Supplementary Fig. 3a). Both VHH#1 and VHH#2 conjugated to AF647 efficiently decorated the bundles and in most cases the individual microtubules could be clearly distinguished when the microtubule centres were 60–70-nm apart (Fig. 1g, Supplementary Fig. 3b). In contrast, when a conventional primary anti-α-tubulin antibody directly coupled to AF647 was used, such bundled microtubules could often not be resolved.

### Comparative analysis of microtubule labels in adherent cells

**Figure 2:**
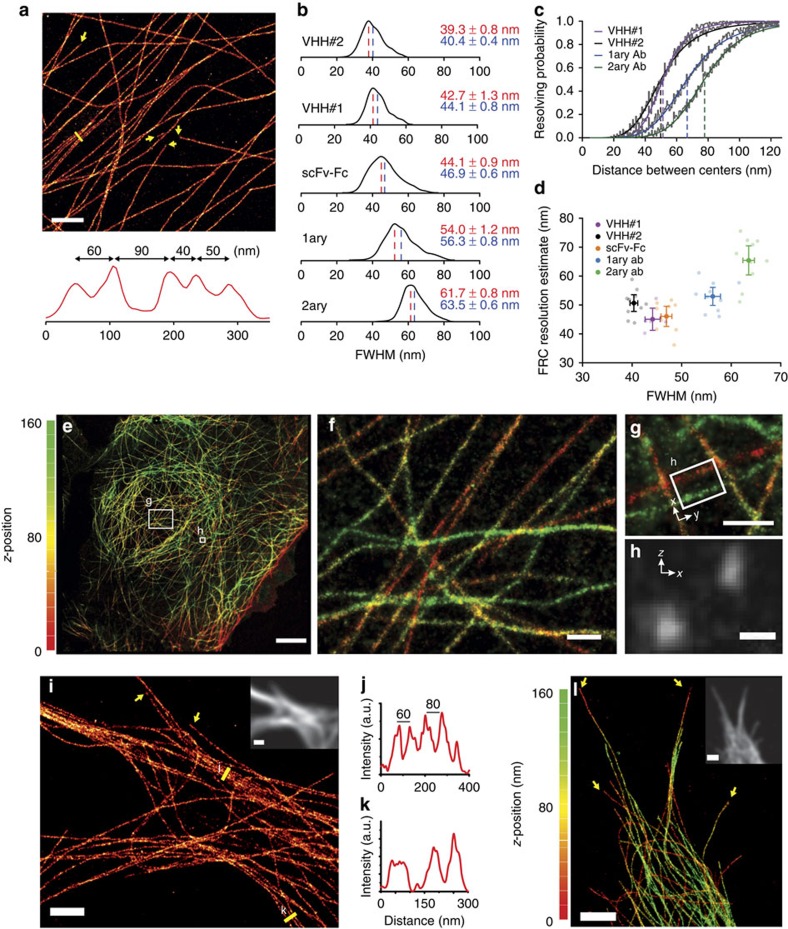
Resolving bundled microtubules in cells using tubulin nanobodies. (**a**) SMLM reconstruction of a Ptk2 cell stained with VHH#1 and intensity profile of closely spaced microtubules along the yellow line. Yellow arrows indicate microtubule ends. Scale bar, 1 μm. A larger field of view of the same cell can be found in Supplementary Fig. 4b. (**b**) Histograms of microtubule FWHM for different probes. scFvs: mixture of human single-chain antibody fragments (scFvs) recognizing α- and β-tubulin. For representative images, see Supplementary Fig. 4a. (From top to bottom: *n*=1,365, 547, 352, 2,462, 2,460 profiles from *N*=10, 5, 9, 10, 10 different acquisitions). Mean (blue) and mode (red) value are indicated±s.e.m. (using *N*). (**c**) Estimation of resolving power for different labels obtained by combining arbitrarily selected line profiles at increasing distance between centres. (**d**) Scatter plot of FRC resolution estimate versus microtubule FWHM for images of microtubules in COS-7 cells stained with different labels. Error bars depict 95% confidence intervals. (**e**) Overview 3D-SMLM reconstruction of a U2OS cell stained with AF647-labelled VHH#1. The z-depth is colour-coded according to the scale on the left of the image. Scale bar, 5 μm. (**f**) Magnified image of the inset in **e**. Colour code is the same as in (e). Scale bar, 500 nm. (**g**) Area containing parallel microtubules at different depth in the cell. Colour code is the same as in **e**. Scale bar, 500 nm. (**h**) Collapsed cross section (z-x) of the volume depicted in **g**. Scale bar, 100 nm. (**i**–**k**) SMLM reconstruction of microtubule bundles labelled with VHH#1 in the dendrites of a hippocampal primary neuron. Yellow arrows indicate microtubule ends and yellow lines were used for line scans across densely packed microtubule bundles **(j**,**k)**. Inset shows the diffraction-limited fluorescence image. Scale bar, 2 μm. **(l)** 3D-SMLM reconstruction of a hippocampal primary neuron labelled with VHH#1. The Z-depth is colour-coded according to the scale on the left of the image. Yellow arrows indicate microtubule ends. Inset shows the diffraction-limited fluorescence image. Scale bar, 2 μm.

When we tested our nanobodies on microtubules in cells, we found that we could resolve microtubules that were spaced down to 40 nm (Fig. 2a). To quantitatively compare the nanobody approach with the other staining methods in cells, we labelled microtubules in fixed Ptk2 and COS-7 cells using different tubulin labels conjugated to AF647. We determined the FWHM from a Gaussian fit to intensity profiles perpendicular to the microtubule averaged over 512-nm length to rule out possible profile artifacts that could arise from low labelling density (Fig. 1b). We found that individual microtubules were densely labelled with the most common diameter (average mode±s.e.m.) varying from 39.3±0.8 nm (VHH#2, *N*=10 data sets with in total *n*=1,365 profiles) to 54.0±1.2 and 61.7±0.8 nm (directly conjugated primary anti-tubulin antibody, *N*=10, *n*=2,462, and primary anti-tubulin+secondary-AF647, *N*=10, *n*=2460, respectively; Fig. 2b, Supplementary Fig. 4a,b; see Methods section for details and Supplementary Fig. 5a for statistical testing). Because in the rendering of the SMLM images we rejected all localizations with localization precision ≥13 nm, these values suggest that fluorophores coupled to primary antibodies are on average ∼12.5-nm displaced from the microtubule lattice (Fig. 1c). Strikingly, this distance is reduced to <2.5 nm for VHH#2.

To translate the observed microtubule FWHM into a resolution estimate, we again analysed composite profiles obtained by superimposing two randomly picked profiles with a set distance between the microtubule centres (Fig. 2c). On the basis of cumulative probability plots obtained for the VHH#1, VHH#2, primary and primary–secondary antibody labellings, ∼50% of all bundled microtubules with 25-nm lattice-to-lattice spacing (corresponding to 50 nm between peaks) will be resolved by the nanobody labels, whereas the directly conjugated primary antibodies or the sandwich labelling will only resolve ∼20% and ∼5% of all microtubule pairs, respectively. Consistent with the *in vitro* bundling results, VHHs are expected to resolve >90% of microtubule pairs with a lattice-to-lattice spacing of 60 nm, which is the typical spacing of tightly bundled microtubules in neuronal dendrites^2^.

To further quantify the gain in resolution, we used the Fourier Ring Correlation resolution measure (FRCrm) as an independent, quantitative estimate of resolution that accounts for both localization precision and probe density^7^. Whereas direct application of the available FRC ImageJ-plugin to our data yielded highly variable results, this could be circumvented by data preprocessing to average different localizations emerging from the same fluorophore emitting over multiple frames (Supplementary Fig. 5b). As expected, smaller apparent diameters also resulted in better FRCrm resolution estimates, with the exception of VHH#2, whose lower labelling density resulted in a worse FRCrm compared to VHH#1, despites its smaller FWHM. For VHH#1, the average FRCrm was 45±4 nm (Fig. 2d). These results demonstrate that our novel anti-tubulin nanobodies provide improved resolution.

### Tubulin nanobody for 3D-SMLM in U2OS cells and neurons

To test how anti-tubulin nanobodies performed in three dimentional (3D)-SMLM, we labelled microtubules in U2OS cells with VHH#1 and performed 3D-SMLM using the biplane approach^8^. We found that microtubules could easily be resolved in *z*-direction (Fig. 2e–h) at distances of 100 nm. Finally, we used the VHH#1 nanobody to perform SMLM on microtubules in primary hippocampal neurons (Days *in vitro*, DIV1) and could successfully resolve individual microtubules in neurites (Fig. 2i–l). The cross-sections across densely packed microtubule bundles indicate a center-to-center spacing of 60 to 80 nm, consistent with earlier results using electron microscopy on cross sections^2^. In several cases, ends of individual microtubules could be clearly identified (Figs 2i,l, arrows). Thus, tubulin nanobodies can be used to resolve neuronal microtubule bundles.

## Discussion

We have introduced novel labels for microtubules that allow using SMLM to resolve previously inaccessible functional details of microtubule organization such as bundling, both *in vitro* and in fixed cells. These labels nicely complement the recently introduced live-cell marker for tubulin^8^ that allows nanoscopy using STED (Stimulated Emission Depletion) microscopy and SIM (Structured Illumination Microscopy) in living cells, but does not remain bound to microtubules upon fixation (Supplementary Fig. 6). Microtubules are key components of many complex cytoskeletal assemblies and their organization, polymerization, motility and interactions with motor proteins are controlled by a plethora of posttranslational modifications and modulating proteins, such as microtubule polymerases, severing proteins and bundlers. Therefore, our ability to resolve individual microtubules in such cytoskeletal assemblies paves the way towards a deeper understanding of the mechanisms underlying microtubule organization and function, both in health and disease.

## Methods

### VHH#1 selection

VHH#1 was selected from a novel library of 3 × 10^9^ humanized nanobodies. Briefly, commercial biotinylated tubulin (Cytoskeleton) was diluted to obtain a 10–20 nM solution (1 ml final) and efficient recovery of biotinylated tubulin was confirmed on 50 μl streptavidin-coated magnetic beads (Dynal). Fractions of bound and unbound samples were compared by western blot using streptavidin–HRP. Adequate amounts of beads and biotinylated antigen were incubated for 2 h with the phage library (10^13^ phages diluted in 1 ml of PBS containing 0.1% Tween-20 and 2% nonfat milk). Phages were previously adsorbed on empty streptavidin-coated magnetic beads to remove nonspecific binders. Phages bound to tubulin-coated beads were recovered on a magnet and washed 10 times (round 1) or 20 times (round 2) using PBS containing Tween-20 0.1%. Bound phages were eluted using 500 μl triethylamine (100 mM) for 10 min. Eluted phages were neutralized using 1 M Tris pH 7.4. Elution was repeated once more. *E*. *coli* (TG1) were infected with the eluted phages. Round 2 was carried out using 10^12^ phages as input. After round 2, 40 bacteria clones were picked at random and used to produce nanobodies in the culture medium. Nanobody specificity was analysed by immunofluorescence as described before^5^ and nanobodies staining microtubules were analysed further.

### VHH#1 expression and purification

For production of VHH#1, WK6 *E*. *coli* containing the plasmid pHEN2–VHH#1–His_6_–cMyc_3_ were grown in 2 l of ‘Terrific Broth' (17 mM KH_2_PO_4_, 72 mM K_2_HPO4, 12 g l^−1^ tryptone, 24 g l^−1^ yeast extract, 0.4% glycerol) containing 2 mM MgCl_2_, 0.1% glucose, and 100 μg ml^−1^ ampicillin with shaking at 37 °C until the *E. coli* had an OD_600_ of 0.6–0.9. Isopropyl β-D-1-thiogalactopyranoside (IPTG) was then added to a concentration of 0.5 mM, and the flasks were shaken at 28 °C overnight (∼16 h). To extract the nanobody from the periplasmic space, cells were centrifuged (5,000*g*, 10 min), resuspended in 24 ml of TES buffer (0.2 M Tris pH 8.0, 0.5 mM EDTA, 0.5 M sucrose) and shaken for 1 h at 4 °C. The cell–TES mixture was then diluted by the addition of 36 ml of TES/4 buffer (50 mM Tris pH 8.0, 0.125 mM EDTA, 0.125 M sucrose), and shaken for 1 h at 4 °C. The cells were then pelleted (5,000*g*, 10 min), and the nanobody-containing supernatant removed. The His_6_-tagged VHH#1 was then purified using HisPur cobalt-agarose resin (Thermo Scientific) following manufacturer's instructions. The eluted protein was concentrated ∼10-fold using ‘Vivaspin' columns (3 kDa MWCO; General Electric). SDS–polyacrylamide gel electrophoresis (PAGE) and Coomassie-staining of the resulting gels revealed the nanobody to be >90% pure. VHH#1 was dialysed overnight against PBS at 4 °C to remove any residual imidazole. The 2 l of culture yielded ∼50 mg of pure nanobody. The stability of VHH#1 was analysed by immunoblotting of a sample stored at 4 °C for >4 months. Two micrograms of VHH#1 were used for Coomassie staining and about 100 ng for immunoblotting using anti-VHH serum 976 (1:2,000 (ref. 9)) or mouse monoclonal anti-c-myc antibody (1:5,000, Abcam) recognizing the carboxy-terminal myc-tag of VHH#1.

### VHH#2 selection

The VHH phage display library was generated from llamas immunized with MCF7 cells^9^. Two rounds of selection were performed as described^9^. For selection of VHHs against tubulin, the bovine brain tubulin (Cytoskeleton) was directly coated onto 96-well NUNC Maxisorp plates (Thermo Scientific) in a series of dilutions (0; 0.1;1; 5 μg in PBS) by incubation for 30 min at room temperature and then overnight at 4 °C. Phages retrieved from the phage-glycerol stock were preincubated with 2% milk-PBS for 30 min at room temperature, and added to the tubulin-coated wells and kept at rppm temperature on a shaker for 2 h. Afterwards wells were washed extensively with 0.05% Tween-20 in PBS. Bound phages were eluted with 100 μl per well of 0.1 M triethylamine followed by recovery via infection of *E. coli* TG1. Phages from the first round were subjected to the second round of selection with 0, 0.1, 1 or 5 μg of coated tubulin. *E. coli* TG1 were infected with the phages from the second selection and plated on LB-agar plates supplemented with ampicillin. Ninety-six random colonies were picked for testing. Expression of VHHs targeted to the bacterial periplasm was induced by addition of 1 mM IPTG at 37 °C overnight. To obtain the periplasmic fraction, bacterial pellets were resuspended in 10 volumes of PBS (pH 7.4) containing protease inhibitor cocktail (Roche), subjected to two freeze/thaw cycles, and spun down for 15 min at 4,600 r.p.m. Periplasm was collected as supernatant fraction. Specificity of VHHs for tubulin was determined by enzyme-linked immunosorbent assay.

### VHH#2 expression and purification

For efficient bacterial expression, four of the most successful and divergent VHH sequences were directly subcloned from pUR8100 into modified pET28a–EPEA vector using SfiI/NotI restriction sites. pET28a–EPEA was created inserting AAACAAAGYQDYEPEA–STOP sequence (NotI/XhoI) in front of the C-terminal 6 × His-myc sequence which allows purification with Capture Select C-tag matrix (Life Technologies). Although all of the constructs were expressed and purified, from now on, we focused on one of the VHH sequences showing the best performance during protein production and labelling (Clone H, that is, VHH#2).

For protein production, an overnight culture of E. *coli* BL21(DE3) transformed with pET28a-VHH#2-EPEA was grown in LB supplemented with kanamycin till OD_600_≈0.8 and induced with 0.5 mM IPTG for 4 h at 25 °C or at 20 °C overnight. VHHs were purified from the periplasmic fraction in PBS (pH 7.4) containing 0.5% Triton-X100, protease inhibitor cocktail (Roche) and 0.5 mM TCEP and purified using Capture Select C-tag matrix according to the manufacturer's instructions (Life Technologies). Bound VHH was eluted from the beads in buffer containing 2 M MgCl_2_, 20 mM Tris-HCL (pH 7.0) and immediately dialyzed against PBS (pH 7.4). Impurities were removed by size exclusion chromatography performed on an ÄKTA FPLC system (ÄKTA purifier, GE Healthcare, UK) using a Superdex 75 gel filtration column. Fractions containing VHH#2 were pooled and upconcentrated to 1–1.5 μg μl^−1^.

### Cell culture and immunostaining

COS-7, MRC5 or Ptk2 cells were plated on 19-mm diameter glass coverslips or 8-well Labtek chambers (Thermo scientific), respectively and cultured in DMEM/Ham's F10 (50/50%) medium supplemented with 10% FCS and 1% penicillin/streptomycin for 2–3 days. Culturing of primary neurons was described before^10^. Briefly, hippocampal primary neurons were prepared from embryonic day 18 rat brains. Cells were plated on coverslips coated with poly-L-lysine (30 μg ml^−1^) and laminin (2 μg ml^−1^) at a density of 40,000 per well. Hippocampal cultures were grown in Neurobasal medium (NB) supplemented with B27, 0.5 μM glutamine, 12.5 μM glutamate and penicillin/streptomycin. For optimal microtubule imaging, cells were pre-extracted and fixed in extraction buffer containing 80 mM PIPES (pH 6.9), 7 mM MgCl_2_, 1 mM EGTA, 0.3% Triton-X100 (Sigma-Aldrich), 150 mM NaCl, 5 mM glucose, 0.25% glutaraldehyde (Electron Microscopy Sciences) for 90 s at 37 °C and then in PBS with 4% PFA and 4% sucrose for 10 min at 37 °C. After fixation, cells were washed two times in PBS and cells were further permeabilized for 10 min in PBS with 0.25% Triton-X100. Cells were then washed three times in PBS, quenched for 10 min with 50 mM NH_4_Cl in PBS, washed again and incubated with Image-IT (Molecular Probes) for 30 min at RT. After three washes with PBS, blocking buffer 1 (used for staining with antibody and VHH#2) containing 2% w/v 2% w/v BSA-c (Aurion)^11^, 0.2% w/v gelatin, 10 mM glycine, 50 mM NH_4_Cl in PBS (pH 7.4) or blocking buffer 2 (used for VHH#1, also works for VHH#2) containing 10% FHS (Gibco, Life Technologies) and 0.1% Triton-X-100 in PBS (pH 7.4) was added for 30–45 min. Primary antibodies or VHHs were diluted in corresponding blocking buffer and were incubated overnight at 4 °C (antibody) or 1–2 days at RT (VHHs). For the secondary antibody labelling, coverslips were washed from the primary antibody and anti-mouse antibody conjugated to AF647 were diluted in a same blocking buffer and added for 1–1.5 h at room temperature. Antibody were α-tubulin (Sigma-Aldrich, clone B-5-1-2, T5168) conjugated to AF647 (dilution 1:100), AF647 conjugated goat anti-mouse IgG (H+L) secondary antibody (Molecular Probes, Life Technologies, dilution 1:500). VHH#1 and VHH#2 were diluted to about 10 μg/ml. All coverslips were extensively washed with PBS shortly before imaging, post-fixed in PBS with 4% PFA and 0.25% GA for 10 min at room temperature and again extensively washed with PBS. For co-staining with F-actin marker, neurons already labelled with VHH#1-AF647 were washed in PBS and incubated with AF568 Phalloidin from Molecular probes (Life Technologies, 1:200 in PBS) for 20 min, extensively washed in PBS and mounted for imaging. For live staining with SiR-tubulin^8^, 100 nM of the probe was added to the growth medium and incubated for 1 h at 37 °C, 5% CO_2_. MRC5 cells expressing plus-end microtubule marker EB3–GFP were used for the life imaging. COS7 cells were fixed with standard pre-extraction/fixation protocol (see above), mixture of 3% PFA and 1% glutaraldehyde for 10 min at 37 °C or 4% PFA for 10 min at 37 °C. Fixed cells were extensively washed in PBS and processed for imaging.

Ptk2 cells were fixed at 37 °C using prewarmed PEM buffer (15 mM PIPES pH 7, 1 mM MgCl_2_, 10 mM EGTA) containing 0.1% Triton X-100 and 0.4% glutaraldehyde for 10 min. They were washed three times with PBS, incubated with PBS containing 50 mM NH_4_Cl for 10 min, washed twice with PBS, incubated with freshly prepared PBS with 0.1 mg ml^−1^ sodium borohydride for 5 min, washed three times with PBS, incubated with Image-IT blocking solution (Life Technologies) for 30 min, washed three times with PBS, and then incubated with blocking buffer 2. Labelled VHH#1 nanobody was then added to a final concentration of 600 nM, and the cells incubated overnight at 25 °C (note that similar labelling was obtained with a 4 h incubation). The cells were then washed three times with PBS containing 0.1% Triton X-100, and twice with PBS and processed for imaging.

### *In vitro* microtubule bundling assay

Rhodamine-labelled microtubuless were prepared from stabilized seeds as described earlier,^12^ and stored at −80 °C. HiLyte Fluor 647-tubulin was purchased from Cytoskeleton and HiLyte Fluor 647-microtubules seeds were made in a same way like Rhodamine–microtubule seeds. The seeds were quickly transferred into a 37 °C water bath, incubated for 5 min and kept in the dark at room temperature for 24 h. Labelled microtubules were diluted 1:30 in PEM80 (80 mM PIPES, pH 6.9, 2 mM MgCl_2_, 1 mM EGTA) containing 10 μM of Taxol (Sigma). Then 50 μl of this dilution was mixed with 0.2 ng of recombinant purified GFP–AtMAP65-1 (ref. 6) and incubated for 20 min at room temperature to allow formation of bundles. Imaging flow chambers were assembled using microscope slides and coverslips connected with double-sided tape. Before each experiment coverslips were plasma cleaned for 10 min, coated for 1 min with 0.4% diethylenetriamine diluted in H_2_O and baked for 1 h at 200 °C. Microtubules with and without GFP–AtMAP65-1 were washed into the flow channels and kept in dark. After 20 min, unbound microtubules were washed out with PEM80 containing 1 μM Taxol. For the immunostainings, attached Rhodamine-microtubules were first fixed for 3 min with 4% PFA and 0.25% GA in PEM80, washed with PEM80 containing 1 μM Taxol, quenched for 10 min with 50 mM NH_4_Cl in PBS, washed again and unspecific binding of proteins to the surface was blocked with blocking buffer 1 for 30 min at room temperature. Samples intended for staining with VHH#1 were in addition blocked with Image-IT for 30 min and then blocked with blocking buffer 2 (see above). Primary AF647-labelled anti-α-tubulin antibody (1:20), VHH#1 (10 ng μl^−1^) or VHH#2 (10 ng μl^−1^) were diluted in corresponding blocking buffer, added to the flow channels and incubated in room temperature for 2 h in the dark. Stained samples were postfixed for 3 min with 4% PFA and 0.25% glutaraldehyde in PEM80, washed with PEM80 and imaged immediately.

### SMLM imaging

Imaging of fixed cells stained with microtubule probes conjugated to AF647 was performed using 10–100 mM mercaptoethylamine (MEA), 5% w/v glucose, 560 μg ml^−1^ glucose oxidase, 40 μg ml^−1^ catalase in PBS. Imaging mixture for *in vitro* microtubule samples contained 100 mM MEA, 5% w/v glucose, 560 μg/ml glucose oxidase, 40 μg ml^−1^ catalase in PEM80 containing 1 μM Taxol.

SMLM microscopy^13^,^14^,^15^ was performed on a Nikon Ti microscope equipped with a 100 × Apo TIRF objective (NA. 1.49), a Perfect Focus System and an additional 2.5 × Optovar to achieve an effective pixel size of 64 nm. Evanescent or oblique laser illumination was achieved using a custom illumination pathway with a 15-mW 405-nm diode laser (Power Technology), a 50-mW 491-nm DPSS laser (Cobolt Calypso), and a 40-mW 640-nm diode laser (Power Technology). Fluorescence was detected using an Andor DU-897D EMCDD camera. All components were controlled by Micromanager software^16^. For SMLM imaging of AF647, the sample was continuously illuminated with 640-nm wavelength light. In addition, the sample was illuminated with 405-nm light at increasing intensity to keep the number of fluorophores in the fluorescent state constant. Typically 5,000–15,000 frames were recorded per acquisition with exposure times of 30–40 ms.

SMLM imaging of Ptk2 cells was performed as described^17^. Imaging chambers were filled with Buffer TN (50 mM Tris-HCl pH 8, 10 mM NaCl) containing 10% glucose, 10 mM MEA (pH adjusted to 8 with KOH; Sigma), 40 μg ml^−1^ catalase (Sigma, C40-100MG), and 0.5 mg ml^−1^ glucose oxidase (Sigma, G2133-50KU), and sealed with a coverslip. Imaging was performed using a standard Nikon NSTORM microscope, using a 647-nm laser adjusted to provide total internal reflection-based illumination. Videos were acquired using an iXon EMCCD (Andor) and a 100 Hz frame rate, with a typical acquisition containing 50,000–100,000 frames. A 488-nm laser was sometimes used to increase the rate at which the AF 647 molecules exited the dark state; however, this was typically not necessary. Acquisitions were then processed to create super-resolution images using custom-written software^3^ (Fig. 2, Supplementary Fig. 4b). For 3D-SMLM, we use the biplane method as described^3^,^18^.

### SMLM localization and rendering algorithms

For Fig. 1 and Supplementary Figs 3 and 4a, we used localization software written in Java as an ImageJ plugin, called Detection of Molecules. Each image in an acquired stack was convoluted with the two-dimensional (2D) Mexican hat kernel matching the microscope's point spread function (PSF) size. The intensity histogram of the convolved image was fitted to a Gaussian distribution and used to calculate the threshold intensity value (mean value of the fit plus three s.d.). The maximum intensity values within individual spots were chosen as initial positions for the peaks' fitting performed on the original image. We used unweighted nonlinear least squares fitting with Levenberg-Marquardt algorithm to the assumed asymmetric 2D Gaussian PSF.

Only fits with a calculated width within ±30% of the measured PSF's standard deviation were accepted. Localizations within one pixel distance in a number of successive frames were considered to arise from the same molecule. In this case the weighted mean was calculated for each coordinate, where weights were equal to inverse squared localization precision. The resulting table with molecule coordinates and precision was used to render the final localization image with 5-nm pixel size for microtubule FWHM analysis, and 10- or 20-nm pixel size otherwise. Each molecule was plotted as a 2D Gaussian with integrated intensity equal to one and with s.d. equal to the localization precision. SMLM-localization and rendering of 3D data into images was done as described before^3^.

### Analysis of super-resolution images

To estimate the FWHM of the microtubules, line region of interests were drawn by hand on the microtubules in the reconstructed image. A custom-made ImageJ macro was then used to generate an intensity profile perpendicular to the region of interests, integrating the intensity values over a length of 500 nm. A Gaussian distribution was fitted to the intensity profile, from which the FWHM was derived as 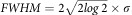.

In order to calculate the probability of separately resolving two microtubule profiles, all profiles used for FWHM calculation were normalized along the *y* axis to an area under the curve of 1 and centred on the *x* axis on the mean derived from the Gaussian distribution fit. To allow for subpixel shifts, bicubic interpolation was applied to the intensity profiles. Two profiles were randomly selected and positioned with their centres a distance between 5- and 125-nm apart from each other. The profiles were summed, and the dip in intensity between the two peaks was calculated. If this dip was >25% of the intensity of the lowest peak, the two profiles were considered to be resolved. After 250 iterations with different randomly selected profiles, the distance between the means was increased by 0.5 nm and the procedure was repeated. At each position, the ratio between resolved and non-resolved sets of intensity profiles was used to calculate the resolving probability. All analysis was performed in the open source software package R.

An independent estimate of image resolution was obtained using Fourier Ring Correlation (FRC), as described previously^7^. In short, particle tables generated by Detection of Molecules were converted to tables with only *x*- and *y*-coordinates for each localization remaining. The FRC plug-in for ImageJ created by the Delft University of Technology Quantitative Imaging Group was then used to obtain a resolution estimate. To obtain consistent results, it was essential to perform frame-to-frame fluorophore linking (see above, Supplementary Fig. 5).

## Additional information

**How to cite this article:** Mikhaylova, M. *et al.* Resolving bundled microtubules using anti-tubulin nanobodies. *Nat. Commun.* 6:7933 doi: 10.1038/ncomms8933 (2015).

## Supplementary Material

Supplementary Information Supplementary Figure 1-6, Supplementary Methods and Supplementary References
