# Supplementary material for: Resolving bundled microtubules using anti-tubulin nanobodies
Source: Nat Commun. 2015 Aug 11;6:7933. doi: 10.1038/ncomms8933 (PMC4918323; doi:10.1038/ncomms8933)
Supplement: Supplementary Information — Supplementary Figure 1-6, Supplementary Methods and Supplementary References [file ncomms8933-s1.pdf]

SUPPLEMENTARY FIGURES

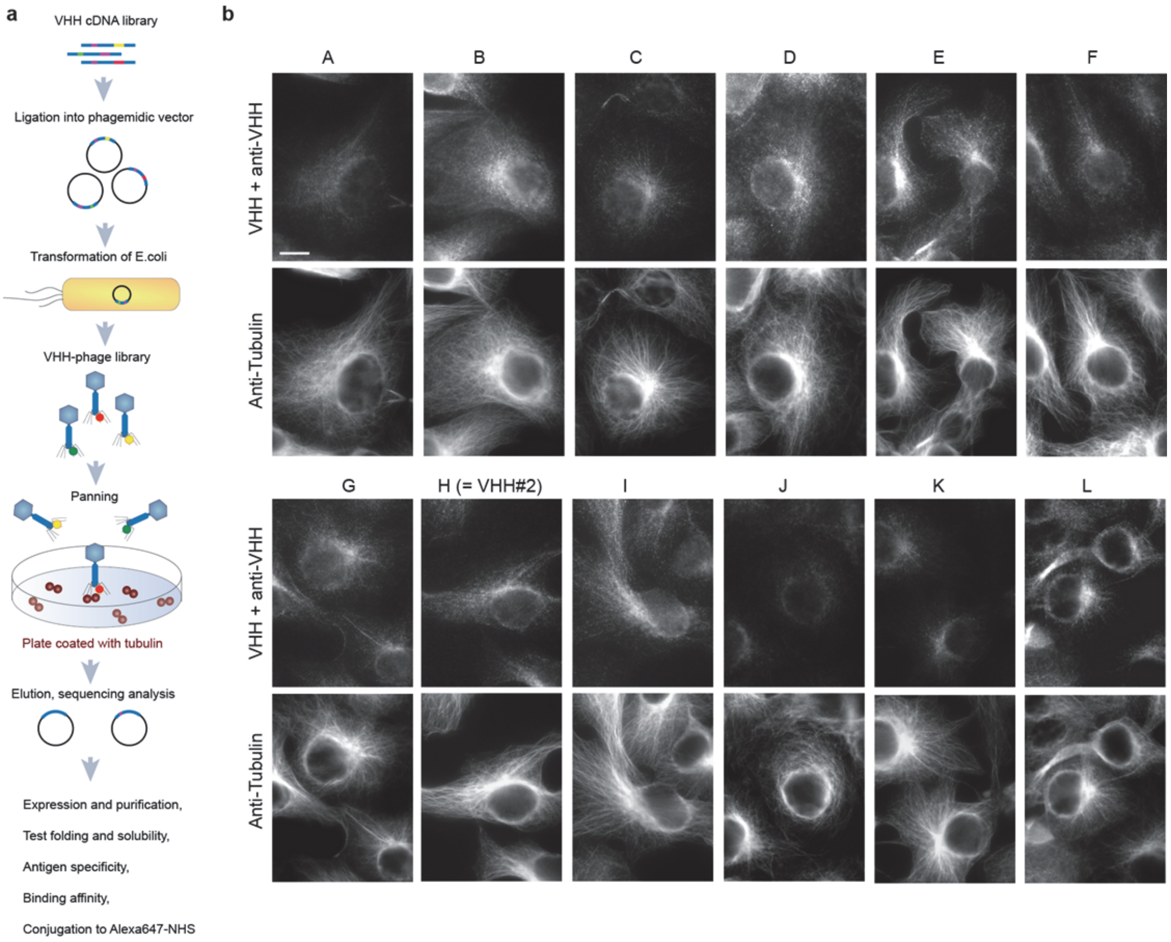

Supplementary Figure 1: Nanobody selection.

(a) Schematic workflow for production of tubulin nanobodies and steps required for selection and quality control of binders. For the details see Materials and methods.

(b) Widefield images of COS-7 cells stained with the periplasm obtained from E. *coli* expressing different VHH sequences (named A-L). VHHs derived from two rounds of panning were tested by ELISA. Then the periplasmic fraction containing the strongest binders was applied on fixed COS-7 cells. Binding of VHHs was detected by immunostaining with polyclonal anti-VHH antibody and by co-localization with microtubules co-stained with mouse anti-α-tubulin antibody. VHH clone H is the same as VHH#2. Scale bars are 5 µm.

Supplementary Figure 2: Biochemical properties of tubulin-binding probes.

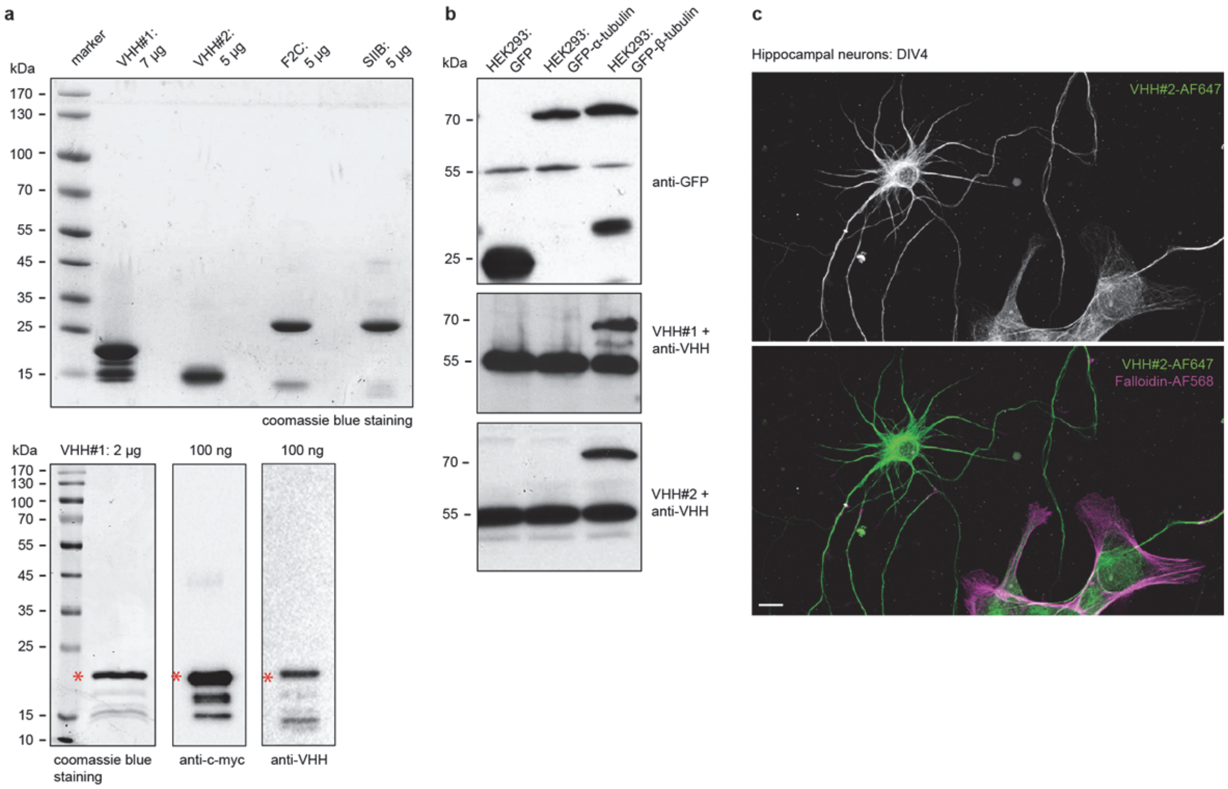

(**a**) Upper panel: Coomassie blue stained SDS-PAGE gel with purified VHH#1, VHH#2 and the antibody fragments F2C and SIIB. The smallest probe VHH#2 migrates at 15 kDa, followed by VHH#1 (20 kDa) and the antibody fragments (about 30 kDa). 5-7 µg of protein were loaded per lane. Lower panel: Coomassie blue stained SDS-PAGE gel with purified VHH#1 stored for more than four month at 4°C and corresponding immunoblots with anti-VHH and anti-myc antibody. Red star indicates intact VHH#1-myc-6xhis band

(**b**) Immunoblot showing that both tubulin nanobodies are directed against beta-tubulin. Lysates were prepared from HEK293 cells transfected with empty GFP plasmid, GFP-α-tubulin or GFP-β-tubulin, and stained with VHH#1, or VHH#2 followed by incubation with anti-VHH rabbit antibody. As a positive control for expression of GFP proteins, the same membranes were developed with anti-GFP rabbit antibody. VHH#1 and VHH#2 recognize the endogenous and overexpressed GFP-β-tubulin, but not GFP-α-tubulin.

(**c**) Confocal image of DIV4 hippocampal neurons stained with VHH#2 directly labeled with AF647 and co-stained with F-actin marker Phalloidin-AF568. Note, that conjugation to AF647-

NHS does not interfere with the epitope binding. Scale bar is 10 µm.

Supplementary Figure 3: Examples of *in vitro* microtubule bundles resolved by dSTORM.

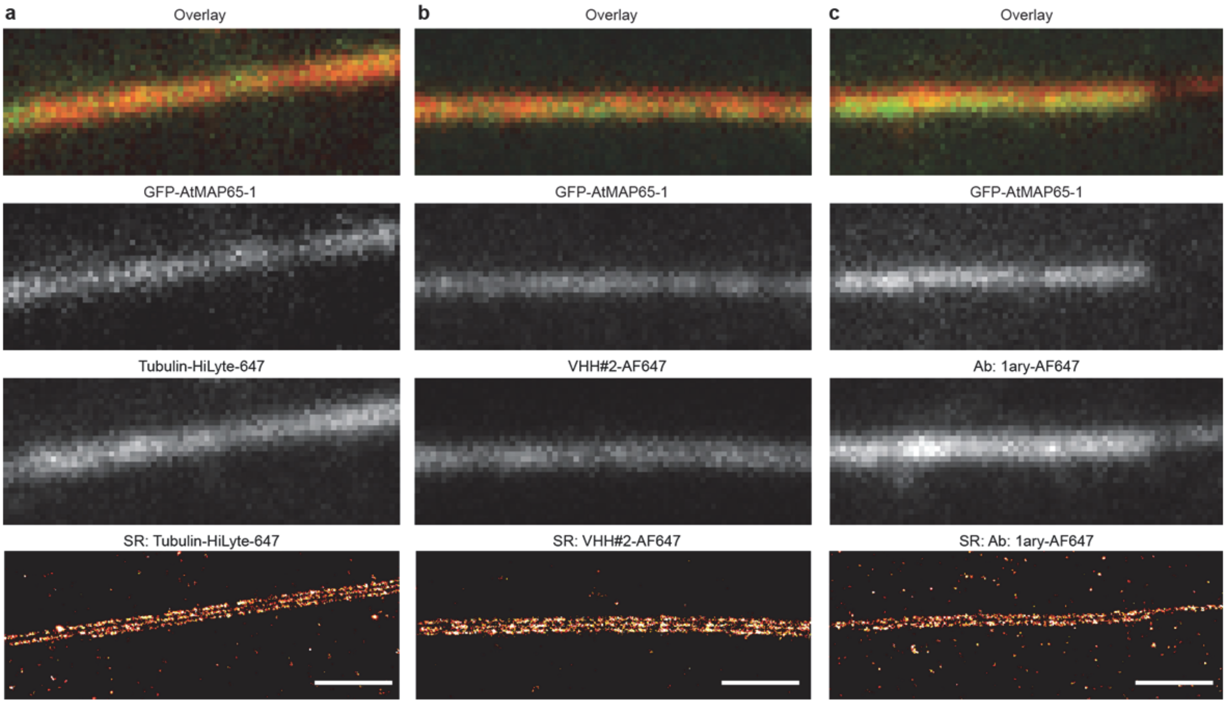

(**a-c**) GFP-AtMAP65-1 bundled microtubules copolymerized with tubulin-HiLyte647 (**a**), or copolymerized with Rhodamine-tubulin (channel is not shown) and labeled with VHH#2-AF647 (**b**) or a conventional primary antibody (1ary) labeled with Alexa647 (**c**). Bottom row shows SMLM reconstructions. Scale bar is 1 µm. Ab stands for antibody.

Supplementary Figure 4: Examples of cellular stainings

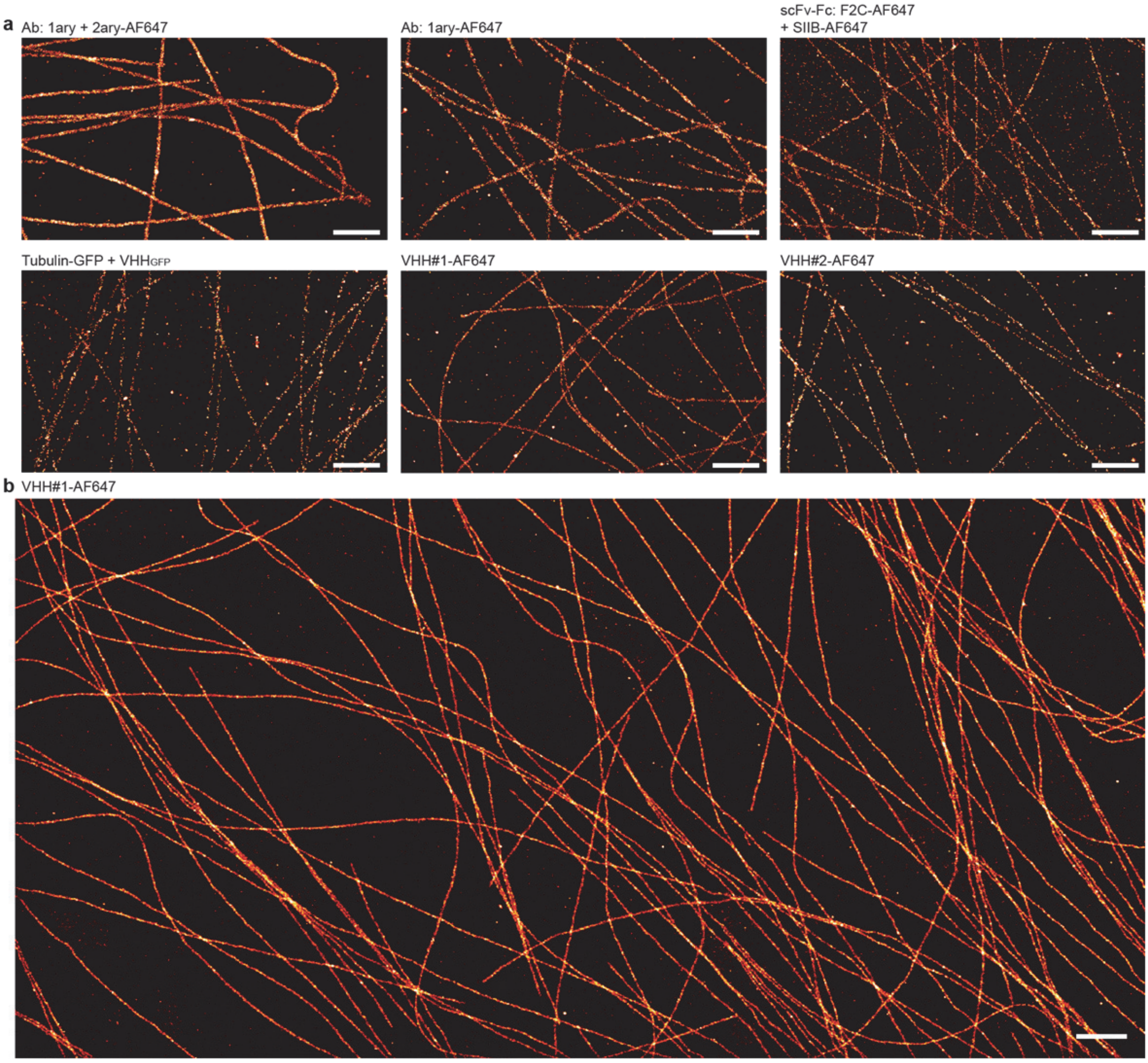

(**a**) Representative images of COS7 cells stained with different labels and quantified in Fig 2e. Scale bars are 1µm. Ab stands for antibody.

(b) Large field of view SMLM reconstruction of microtubules in a Ptk2-cell (also shown in Fig. 2d). Scale bar is 1µm.

Supplementary Figure 5: Significance and FRC correction effects

a

MEAN				
	VHH#1	VHH#2	scFV-Fc	1ary Ab
VHH#2	NS			
scFv-Fc	NS	<0.005		
1ary Ab	<0.005	<0.005	<0.005	
2ary Ab	<0.005	<0.005	<0.005	<0.005
MODE				
	VHH#1	VHH#2	scFV-Fc	1ary Ab
VHH#2	NS			
scFv-Fc	NS	<0.01		
1ary Ab	<0.005	<0.005	<0.005	
2ary Ab	<0.005	<0.005	<0.005	<0.005

b

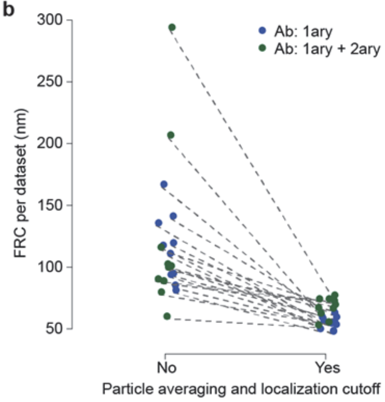

(a) p-values for comparing the mean values and mode values. This was tested using the Welch Two Sample t-test (two-sided), and using the Bonferroni correction for testing of multiple conditions.

(b) Effect of frame-to-frame fluorophore linking on the FRC resolution estimate.

Supplementary Figure 6: SiR Tubulin does not remain bound to microtubules upon fixation.

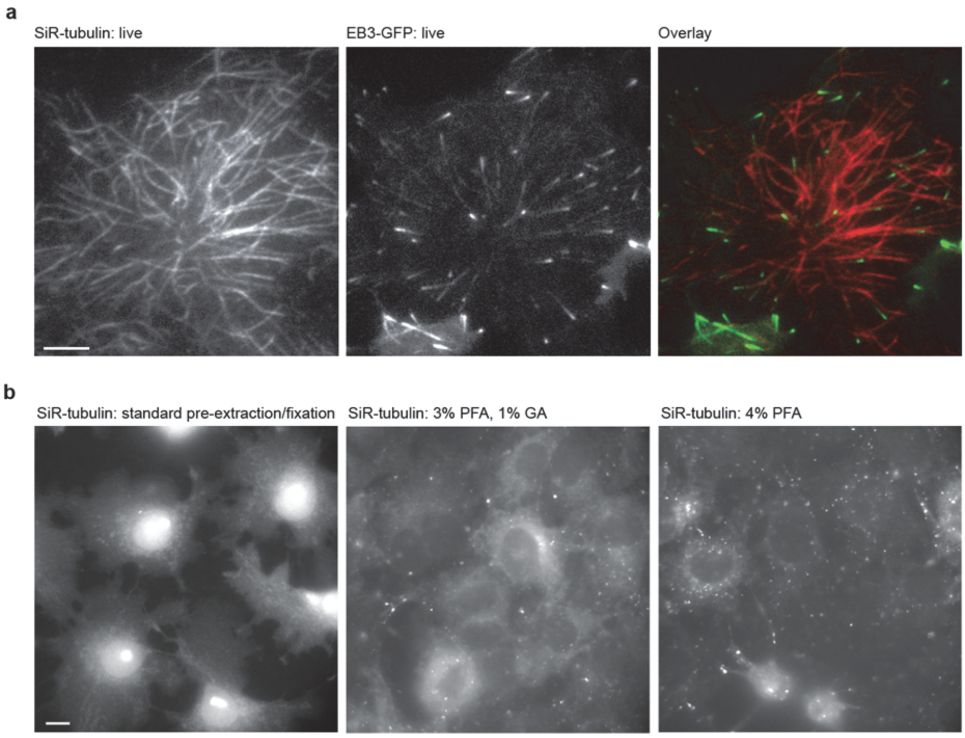

(a) Still image from a live-cell recording of a MRC5 cells expressing EB3-GFP and incubated with SiR tubulin.

(b) Example images of cells incubated with SiR tubulin before fixation using the indicated protocol. Scale bars are 5 µm. GA stands for glutaraldehyde.

SUPPLEMENTARY METHODS

Purification and labeling of the antibody fragments F2C and SIIB

Human-derived single chain variable fragments (scFvs) were previously selected and characterized [1]. The antibody fragments were cloned in a custom designed plasmid (His_{14x}-ZZ-SUMO-F2C or –SIIB) and transformed into SHuffle E. *coli* strain for cytoplasmic expression. Bacteria were grown at 20°C in Terrific Broth medium containing 4 mM $MgCl_2$, and 100 µg/ml kanamycin until OD_{600} reached ~1.5. Expression was induced with 0.5 mM IPTG. Flasks were shaken at 160 rpm for ~16 h at 20°C. EDTA was added to a final concentration of 0.5 mM and cultures were finally centrifuged at 6000 x g for 10 min. Bacterial pellets were resuspended in binding buffer (50 mM HEPES, 500 mM NaCl, 5 mM $MgCl_2$, 20 mM imidazole, 10% glycerol) supplemented 0.25 mM of PMSF before disruption by sonication on ice. Suspension was centrifuged for 60 min at 12.000 g and 4°C. Supernatants were filtered (0.45 µm pore size) and incubated for one hour at room temperature with complete His-Tag purification resin (Roche) followed by several washes as recommended by the supplier. Elution was achieved using 500 mM imidazole in binding buffer. To remove the histidine-tag, His_{14x}-ZZ-SUMO-scFvs proteins were incubated for 1 hour at room temperature with yeast-derived His_{6x}-SUMO protease (Ulp1). After specific cleavage of the SUMO-domain, antibody fragments were desalted and passed through a HisTrap 1 ml column using Äkta HPLC system to remove unwanted cleaved fragments and His_{6x}-SUMO protease. The high purity (>90%) and the untagged nature of purified scFvs were analyzed via SDS-PAGE. Antibody fragments were conjugated to AF647 using succimidyl ester chemistry. The stock of fluorophore was dissolved in anhydrous DMSO at a final concentration of 10 µg/µl. The coupling reaction was performed in freshly prepared 100 mM $NaHCO_3$ buffer (pH 8.0). The reactive fluorophore was added in 6 fold molar excess to the scFvs containing solution while mixing for 1.5 h at room temperature and protected from light. Unconjugated reactive fluorophores were quenched by adding an excess of hydroxylamine (~150 mM) with subsequent mixing for 15 minutes at room temperature. Antibody fragments were efficiently separated from uncoupled dye molecules using a 14 cm column (BioRad) packed with superfine G25 sephadex resin. Elution fractions were tested by immunostainings, the best fractions were pooled and stored at 4°C adding to 0.05% sodium azide or in 50% glycerol at -20°C.

ELISA assay for determining specificity of VHHs for tubulin

A flat-bottom 96-well plate (Maxisorp) was coated with 0.1 µg of bovine brain tubulin, washed with PBS and pre-blocked with 2% milk-PBS for 30 min at RT. 10 µl of periplasm fractions in 100 µl 2% milk-PBS was added to the wells and incubated for 90 min at RT. The plate was washed twice with PBS and the primary rabbit polyclonal anti-VHH antibody ([2], 1:2000) was added for 1 h at RT in 2% milk-PBS. Plate was again washed twice with PBS and incubated with secondary donkey anti-rabbit-PO antibody (Dako, 1:5000) for 1 h at RT. After final washing steps, 100 µl/well OPD+H_2O_2 was added and the plate was incubated for 30 min at RT. The reaction was stopped with 50 µl/well of H_2SO_4 and relative VHHs presence was analyzed by measuring optical density at λ= 490 nm with an Ultramark spectrophotometer (Biorad). Monoclonal anti-α-tubulin antibody (Sigma) was used as positive control. 14 colonies showing the strongest signal were selected for further study and VHH DNA was isolated and sequenced using an M13 reverse primer.

Determination of the tubulin subunit detected by VHH#1 and VHH#2.

HEK293 cells were plated on 10 cm^2 dishes and cultured in DMEM/Ham's F10 (50/50%) medium supplemented with 10% FCS and 1% penicillin/streptomycin for 2 days. Cells were transfected using MaxPEI (PolyEthylenImine, Sigma) with 10 µg of pDNA encoding for empty EGFP, β-tubulin(2C)-EGFP or α-tubulin-EGFP in β-actin vectors and grown for 48h at 37°C and 5% CO_2. Following transfection, dishes were washed with 1 ml of PBS and extracted with 500 µl extraction buffer (PBS pH 7.4, 1% TritonX-100, 2mM Ca^{2+}, protease inhibitor cocktail). Cell suspensions were frozen at -80°C, thawed, incubated on ice for 2 h, and finally spun down at 13.000 rpm for 15 min. Obtained supernatants samples were loaded on 12% SDS-PAGE gels, and transferred for 90 min at 15V via semi-dry western blotting onto a PVDF membrane. The membrane was blocked with 5% milk-PBS for 1h, and incubated with VHH#1 or VHH#2 (2 µg/ml) for 4h at RT on a shaker. After PBS wash, membranes were incubated in either home-made primary rabbit anti-VHH serum 976 (1:2000 in milk-PBS; [2]) or rabbit anti-GFP antibody (1:5000 in PBS; Abcam) overnight at 4°C. Next day, another cycle of PBS wash was performed, and HRP-conjugated secondary goat anti-rabbit antibodies (Dako) were added for 60 min at RT, blots were extensively washed and processed for developing.

Conjugation of fluophores to antibodies and VHHs

Conjugation of AF647 (Alexa Fluor® 647 Carboxylic acid, Succiminidyl Ester, Molecular Probes, Life technologies) to monoclonal anti-α-tubulin antibody (Sigma) was performed according to the protocol described before [3,4]. Some modifications were introduced for labeling of VHH. Conjugation reaction was performed in freshly prepared 100 mM $NaHCO_3$ buffer (pH 8.0) where the ratio of VHH:fluorophore was 1:5 for VHH#1 and 1:3 for VHH#2. Samples were incubated for 1-6 h at RT in the dark and labeled VHH was separated from non-conjugated dye by passing through a NAP25 column (GE Healthcare). Eluted VHH-AF647 was upconcentrated to 0.5-0.7 µg/µl, labeling efficiency was measured as described before [3]. The absorption spectrum of the preparations revealed a labeling ratio of 1.5 fluorophores per VHH#1 and 0.3 fluorophores per VHH#2. Sodium azide was then added to the mixture to a final concentration of 0.05%, and the proteins were stored at 4°C.

Microtubule labeling simulations

Generative model

Images of *in silico* microtubules were synthesized from a generative 3D model. First the main axis (backbone) of straight microtubule with a specified length was marked. The backbone segment was populated with tubulin heterodimers forming a regular 25 nm diameter 3-start helix consisting of 13 straight protofilaments. The coordinates of tubulin subunits were spaced 8 nm apart from each other along the protofilament.

Tubulin subunits were randomly labeled with fluorophores to achieve the desired probe density. If a tubulin molecule was marked as labeled, the fluorophore coordinates were generated and added to the model. The position of the fluorophore was determined by addition of the offset distance m extending outwards from the surface (i.e. in the radial direction) to the coordinates of the corresponding tubulin. A small rotation by angle φ (uniformly distributed on the interval from 10° till -10°) in the plane perpendicular to the microtubule axis was added to simulate flexibility in the binding of the antibody probe to the antigen.

To simulate widefield images, the positions of fluorophores were projected onto a 2D plane and convoluted with a 2D isometric Gaussian kernel with a standard deviation equal to that of the point spread function of the microscope. This produced a continuous 2D distribution of intensity. Further the intensity was integrated over the area of each pixel of the future image. The final pixel intensity value was drawn from the Poisson distribution with the mean equal to the integrated intensity value from the previous step, to account for the shot noise of light registration.

Simulated super-resolution microscopy images of microtubules were generated using the same rendering algorithm as for the real SMLM dataset reconstruction (see above). To account for the uncertainty in the localization of fluorescent signal during "virtual" SMLM acquisition, each fluorophore position was shifted by random displacement of magnitude d drawn from a Gaussia localization accuracy distribution with mean ± SD of 7.5±2.5 nm. If the displacement magnitude d was above a specified precision cutoff threshold t, its random value is generated again until it is below the threshold, so the probe density remains constant. An angle of displacement direction θ was drawn from a uniform random distribution covering the full 360° for each of the fluorophores. The new position of the fluorophore was used to render its image.

Dataset

The generative model was transcribed into a Matlab code and run in GNU Octave version 3.8.2 on Mac OS X 10.10.0, Intel Core i7 2.8 GHz, 16GB RAM. A dataset of images was generated where the model parameters (probe density, position of the probe and precision cutoff) varied in a wide range. The values for probe density were chosen at 0.1%, 0.3%, 1%, 5%, 7%, 10%, 15%, 30%, 50%, 75% and 100%. The values for mean probe position were chosen at 0.0, 2.5, 5.0, 7.5, 10.0 and 12.5 nm. The values for the precision cutoff threshold were chosen at 3, 5, 8, 10, and 13 nm. For each combination of the three parameters a microtubule with a contour length of 80 μm was generated. The generated microtubule was centered within the field of view before being rendered into a widefield and super-resolution image. The widefield images were generated with a pixel size of 64 nm and the standard deviation of microscope's point spread function equal to 1.8 pixels (=115.2 nm). The super-resolution images were generated with a pixel size of 4 nm. All microscopy images were exported to local storage in 16-bit gray scale TIFF image format.

Intensities were uniformly scaled prior to export to take advantage of the full dynamic range of the image format.

Analysis of simulated images

The super-resolution images were sampled with a window 512 nm long (in axial direction) and 400 nm wide. The window was placed at non-overlapping intervals along the entire microtubule, resulting in 150 samples per microtubule. Empty regions (i.e. summed intensity of zero) were rejected from the analysis. This situation was particularly common for the low (<3%) probe density. An intensity line profile was derived from a sampled region by averaging the intensities along the axial direction. A 1D Gaussian function with four parameters (background level, peak amplitude, peak position, and peak width) was fitted to the intensity line profile using a Levenberg-Marquardt nonlinear regression algorithm. The regression algorithm was run for a maximum of 1000 iterations or until convergence was achieved with an accuracy of 10^{-3}. The mean and standard error of the FWHM were computed for each condition in the dataset. The FWHM was calculated from the fitted peak width as described. The analysis of the resolving probability of two microtubules was performed as described above.

SUPPLEMENTARY REFERENCES

1 Nizak, C. *et al.* Recombinant antibodies against subcellular fractions used to track endogenous Golgi protein dynamics in vivo. *Traffic* **4**, 739-753 (2003).

2 Kijanka, M. *et al.* Rapid optical imaging of human breast tumour xenografts using anti-HER2 VHHs site-directly conjugated to IRDye 800CW for image-guided surgery. *European journal of nuclear medicine and molecular imaging* **40**, 1718-1729 (2013).

3 Cloin, B. M., Hoogenraad, C. C., Mikhaylova, M. & Kapitein, L. C. in *Immunocytochemistry and Related Techniques* Vol. 101 (eds A Merighi & L Lossi) 389-408 (Springer, 2015).

4 Yau, K. W. *et al.* Microtubule minus-end binding protein CAMSAP2 controls axon specification and dendrite development. *Neuron* **82**, 1058-1073 (2014).
